# Communication competence, self-efficacy, and spiritual intelligence: evidence from nurses

**DOI:** 10.1186/s12912-023-01262-4

**Published:** 2023-04-06

**Authors:** Gholamhossein Mehralian, Ali Reza Yusefi, Neda Dastyar, Shima Bordbar

**Affiliations:** 1 Nottingham Business School, Nottingham, UK; 2 Department of Public Health, School of Health, Jiroft University of Medical Sciences, Jiroft, Iran; 3 Department of midwifery, Nursing and Midwifery School, Jiroft University of Medical Sciences, Jiroft, Iran; 4 Health Human Resources Research Center, School of Health Managemet and Information Sciences, Shiraz University of Medical Sciences, Shiraz, Iran

**Keywords:** Communication, Self-efficacy, Spirituality, Intelligence, Nurses, Hospitals

## Abstract

**Introduction:**

Communication skills and acceptable levels of spiritual intelligence (SI) are the prerequisites of the nursing profession, which can significantly impact the individual and organizational performance of nurses. This study aimed to investigate the competency and self-efficacy of communication and its relationship with the SI of nurses.

**Methods:**

This cross-sectional study was conducted in 2021 and included 312 nurses working in a COVID-19 hospital in the south of Iran. The data collection instruments were the Standard Communication Competence Scale, Communication Self-Efficacy, and SI Questionnaires. Data were analyzed with SPSS software version 23 using descriptive and inferential statistics, and t-test, ANOVA, Pearson’s correlation coefficient, and multiple linear regression were performed at 5% significance level.

**Results:**

The mean scores of nurses’ communication competence, communication self-efficacy, and SI were 89.11 ± 7.32 out of 180, 64.45 ± 5.61 out of 120, and 147.13 ± 11.26 out of 210, respectively. A direct and significant correlation was observed between competence (*r* = 0.527, *p*<0.001) and communication self-efficacy (*r* = 0.556, *p*<0.001) with spiritual intelligence. The dimensions of spiritual intelligence, including the ability to deal with and interact with problems, self-awareness, love and affection, general thinking and doctrinal dimension, and dealing with moral issues, were identified as predictors of nurses’ communicative competence and self-efficacy (*p*<0.05). There was a positive and significant correlation between nurses’ competence and self-efficacy with their age (p<0.05). The nurses’ mean communication competence and self-efficacy score were different regarding their level of education and the number of shifts (p<0.05). The mean scores of self-efficacy revealed a statistically significant difference between the participants’ gender and the number of patients under observation (p<0.05). Moreover, the nurses’ SI significantly correlated with age, and the mean scores of this intelligence had statistically significant differences regarding gender (p<0.05).

**Conclusion:**

The nurses’ communication competence and self-efficacy were at a moderate level. Considering the correlation and predictive role of SI and its dimensions, it is recommended to promote problem-solving skills, improve self-awareness, and pay attention to moral standards to nurture communication competence and self-efficacy among nurses.

## Introduction

Communication plays a key role in the nursing profession and caring for patients and is of paramount importance in detecting and meeting patient needs. Nurses must have competence, efficiency, and special communication abilities [1] because nurses face many challenges in daily treatment environments, the severity, and impact of which vary by the type of treatment environment [2, 3]. Communication competence is one of the tools to promote the efficiency of nursing services [4]. It is confirmed as a valuable resource for improving nursing services, and nurses’ communication competence is of the essence to establishing a therapeutic relationship with patients and is considered a professional value [5]. Previous studies have indicated that communication skills and acceptable communication with nurses can help and improve the patient’s health status regarding the disease and his physical, emotional, mental, and social status [6]. Furthermore, communication competence and the ability to establish effective communication increase nurses’ awareness of patient issues and problems [7], improve decisions about the discharge and transfer of patients [8], provide appropriate health solutions, and promote the quality of patient care [9], enhances the sense of participation and cooperation in the treatment team, promotes skills, knowledge, and attitude, decreases treatment errors [10], reduces the length of hospital stay [11], lowers treatment costs, reduces stress and job burnout, increases productivity and satisfaction, and promotes job performance [9].

On the other hand, communication disorders lead to long hospital stays, increased missed care, and errors [12]. Some studies have documented that many public complaints and the incorrect application of orders by the patient are not the results of the incompetence of health care workers, including nurses; however, they are rooted in communication problems, which also lead to patient dissatisfaction [11, 13, 14]. Accordingly, the communication competence of nurses who are in direct contact with patients is of great importance, and its promotion is necessary as a preventive intervention since poor communication competence leads to threats and disturbances in patient safety [15]. There is a need to examine and consider communication competence as an essential component for success in nursing services and for promoting job performance [4].

One of the factors associated with communication competence is communication self-efficacy [5]. All nurses are expected to be competent in communication, implying that they should communicate effectively with their patients, families, and colleagues [16]. Self-efficacy refers to confidence a person has in himself by mastering the desired skills [17]. This concept reflects an individual’s ability to cope with many stressful situations effectively [18] and has many effects on stress, job burnout, job satisfaction, and performance [19]. Self-efficacy in nursing mediates knowledge and practice and plays a key role in applying scientific and professional knowledge and skills [20]. Individuals with high self-efficacy are more likely to use their motivation and cognitive resources to perform the actions needed to achieve the goal [20]. According to previous studies, nurses’ self-efficacy and ability to communicate effectively correlate with favorable outcomes for patients [21], increased patient safety, and improved outcomes for nurses [22]. Accordingly, self-efficacy evaluation needs to be further considered.

From another perspective, nursing is one of the professions in which the role of multiple intelligences, including spiritual intelligence (SI), is more highlighted than in other health professions [23]. The concept of spiritual intelligence involves seeing life as an interconnected system, which leads to the identification and organization of a person’s skills and capabilities in a way that facilitates high adaptability and self-mastery [24]. This intelligence has different dimensions, including general thinking and doctrinal dimension, the ability to deal with and interact with problems, self-awareness, love, and affection, and dealing with moral issues [25].

SI is of interest not only in the individual fields of nursing but also in organizational fields [26]; hence, one of the reasons for increasing research on spirituality can be its remarkable impact on improving individual and organizational performance [27]. Using their spiritual intelligence, nurses can solve problems, give meaning to their work and activities, become aware of the meaning of their performance, and find out their more valid actions [28]. The effect of spiritual intelligence on the self-efficacy of nurses [29–33] and their competence acquisition in this field [34–36] is a topic that has been mentioned in previous studies. This intelligence, due to the use of the internal resources of the individual to increase the capacity of attention, tolerance, and adaptability, can help to develop the understanding of nurses in the field of personal and work communication and to improve the level of their competence and relational self-efficacy [37]. A nurse with high spiritual intelligence recognizes the pain and suffering of others, listens impartially, accepts differences, recognizes various human values, refuses immoral options, and understands others [38, 39]. Therefore, one of the factors that may affect the communication competence and self-efficacy of nurses is spiritual intelligence. As a necessity, communication is the most critical factor in the development and excellence of nurses to perform the best operations. As spiritual intelligence has a crucial impact on nurses’ communication performance, this study will examine its impact on nurses’ communication competence and self-efficacy.

## Methods

### Design and setting

From June to September 2021, a descriptive-analytical and cross sectional study was conducted in the Hazrat Ali Asghar (AS) Hospital in Shiraz, south of Iran. AS Hospital has 200 beds, two general treatment and administrative departments. The treatment section include 11 departments, including acute events one (34 nurses in three shifts), acute events two (30 nurses), four ICUs (with totally 126 nurses), surgery (22 nurses), internal neurology (28 nurses), internal medicine for men (36 nurses), and internal medicine for women (36 nurses).

### Participants

The research population encompasses the nurses working in the AS hospital. In this study, the census method was used, and all nurses (n = 312) participated in the study. Employed in different clinical wards of a hospital was the inclusion criterion. The exclusion criteria were employment in non-clinical wards such as the administrative and financial wards of the hospital.

### Instruments

The data collection tool encompassed a four-section questionnaire. The first section of the questionnaire addressed the nurses’ demographic information (namely age, work experience, gender, marital status, level of education, type of employment, number of shifts per month, and number of patients under observation per work shift). The second section was the communicative competence scale (CCS) developed by Wiemann [40]. This section contained 36 items classified and scored on a five-point Likert scale ranging from “strongly disagree” ( = 1) to “strongly agree” ( = 5) for items with positive connotations. The items with negative concepts were reversely scored. To determine the overall communication competence, poor (36–72), moderate (73–108), acceptable (109–144), and excellent (145–180) categories were used [41]. The validity and reliability (with Cronbach’s alpha coefficient = 0.96) of this scale were confirmed by Wiemann [40].

The third section was the communication self-efficacy questionnaire (SEQ) developed by Axboe et al. [42]. This section consisted of 12 questions classified and scored based on a 10-point scale ranging from “not sure at all” ( = 1) to “Highly sure” ( = 10). To determine the overall communication self-efficacy, poor [12–39], moderate (40–66), acceptable (67–93), and excellent (94–120) categories were used [41]. Axboe et al. confirmed the validity and reliability of SEQ (with Cronbach’s alpha coefficient of 0.95) [42].

Finally, the fourth section was the SI questionnaire developed by Kazdin et al. This section of the questionnaire contained 42 items in four dimensions: general thinking and doctrinal dimension (12 items), the ability to deal with and interact with problems (15 items), self-awareness, love, and affection (8 items), and dealing with moral issues (7 items). The items in this section were scored on a Likert scale from never ( = 1), rarely ( = 2), sometimes ( = 3), often ( = 4), and always ( = 5). Questions with negative connotations were scored reversely. Regarding the scores, the general state of SI was categorized as poor (42–84), moderate (85–126), acceptable (127–168), and excellent (169–210) [25]. The validity and reliability of this section of the questionnaire were confirmed by Rani et al. (Cronbach’s alpha coefficient = 0.95) [25].

Although the validity and reliability of the communication competence, communication self-efficacy, and SI questionnaires had been confirmed in previous studies [25, 40, 42], the validity and reliability of this questionnaire were also measured in this study. To this end, the validity of the questionnaire was assessed by 10 members of the academic staff and experts in the field of health and treatment management from Iran’s universities of medical sciences. In this regard, the calculated Content Validity Index (CVI) and Content Validity Ratio (CVR) were 0.88 and 0.91 for the communication competence scale, 0.86 and 0.89 for the communication self-efficacy questionnaire, and 0.84 and 0.87 for the SI questionnaire, respectively. To measure the reliability of the questionnaire, a sample of 30 nurses was pre-tested, and Cronbach’s alpha coefficients were 0.94 for the communication competence scale, 0.92 for the communication self-efficacy questionnaire, and 0.88 for the SI questionnaire; hence, the reliability of the questionnaire was confirmed.

### Procedures and statistical analysis

Two researchers (SB and ND) visited the hospital on different weekdays in morning, evening, and night shifts to collect the required data and then distributed and collected questionnaires. To observe the ethical considerations, nurses participated in the study and completed the questionnaires voluntarily. After explaining the research objectives to the participants, the confidentiality of the responses was emphasized, and informed consent was obtained from all the nurses. Then the questionnaires were distributed to the nurses in person and collected on the same day. A questionnaire was distributed to nurses at their workplace and collected the same day. The average time to complete each questionnaire was 30 min.

In the next phase, the collected data were imported to SPSS software version 23. Pearson’s correlation coefficient was used to investigate the correlation between nurses’ communication competence and communication self-efficacy with their SI and the correlation of these three variables with nurses’ age and work experience. T-test was used to examine the differences in the mean scores of the three main research variables regarding gender, marital status, and level of education. ANOVA test was also run to detect differences in the mean scores of nurses’ communication competence, communication self-efficacy, and SI regarding the type of employment, the number of shifts, and the number of patients under observation. Finally, multiple linear regression was used to investigate the simultaneous effect of different dimensions of SI on nurses’ communicative competence and self-efficacy.

## Results

The nurses’ mean age was 31.32 ± 7.18 years, with most participants (53.20%) in the age group of < 30 years. The average work experience was 6.24 ± 6.38 years, with most nurses (71.47%) in the group of < 10 years. Moreover, 65.06% of the participants were female, and the others were male. Most of the respondents had a bachelor’s degree (88.46%), a project workforce (58.34%), and a history of 10–20 shifts per month (45.84%). For most of the nurses, the number of patients under observation per shift was > 3 patients (83.98%) (Table 1).

**Table 1:** Frequency distribution of nurses (n=312)

Variable	Categories	Frequency	Percentage
Age (years)	30> 30–40 40<	166 127 19	53.20 40.71 6.09
**Total**	------	312	100
Work experience (years)	10> 10–20 20<	223 76 13	71.47 24.36 4.17
**Total**	------	312	100
Gender	Male Female	109 203	34.94 65.06
**Total**	------	312	100
Marital status	Single Married	69 243	22.12 77.88
**Total**	------	312	100
Level of education	Bachelors’ Master’s	276 36	88.46 11.54
**Total**	------	312	100
Type of employment	Formal Contractual Contract-Based Project Corporate	82 7 19 182 22	26.28 2.24 6.09 58.34 7.05
**Total**	------	312	100
Number of shifts per month	10> 10–20 20<	28 143 141	8.97 45.84 45.19
**Total**	------	312	100
Number of patients under observation in each work shift	2 3 3<	7 43 262	2.24 13.78 83.98
**Total**	------	312	100

The mean and standard deviation of communication competence was 89.11 ± 7.32 out of 180, indicating a moderate level. Among the communication competence items, the largest and the smallest mean and standard deviation scores were obtained for the following items: “S generally knows what type of behavior is appropriate in any given situation.“ (2.76 ± 0.82 out of 5) and “S is not afraid to speak with people in authority.“ (2.19 ± 0.36 out of 5), respectively. The mean and standard deviation of communication self-efficacy was 64.45 ± 5.61 out of 120, indicating a moderate level. Among the communication self-efficacy items, the largest and the smallest mean and standard deviation scores were obtained for the following items: “Using appropriate non-verbal behaviors” (5.53 ± 1.76 out of 10) and “Making a pre-prepared plan to talk to the patient” (5.24 ± 1.47 out of 10), respectively.

The mean and standard deviation of SI was equal to 147.13 ± 11.26 out of 210, indicating an acceptable level. The mean and standard deviation was 42.24 ± 4.71 out of 60 for general thinking and doctrinal dimension, 52.99 ± 5.01 out of 75 for the ability to deal with and interact with problems, 27.38 ± 3.86 out of 40 for self-awareness, love, and affection, and 24.52 ± 3.58 out of 35 for dealing with moral issues. According to Table 2, there was a statistically significant correlation between the nurses’ communication competence and communication self-efficacy with their SI (p < 0.05).

**Table 2:** Coefficients of correlation between nurses’ communication competence and self-efficacy with their SI

Variable	Spiritual Intelligence (SI)				
	General thinking and doctrinal dimension	The ability to deal with and interact with problems	Self-awareness, love, and affection	Dealing with moral issues	**Total SI**
**Communication competence**	*r* = 0.459 *p* = 0.002	*r* = 0.674 *p* < 0.001	*r* = 0.583 *p* < 0.001	*r* = 0.442 *p* = 0.004	***r*** **=** **0.527** ***p*** **< 0.001**
**Communication self-efficacy**	*r* = 0.566 *p* < 0.001	*r* = 0.621 *p* < 0.001	*r* = 0.602 *p* < 0.001	*r* = 0.391 *p* = 0.005	***r*** **=** **0.556** ***p*** **< 0.001**

To determine the simultaneous effect of different dimensions of SI on the communication competence and communication self-efficacy of nurses, the results of multiple linear regression analysis showed that the significant variables in the model determined by the Enter method, in order of importance, were as follows: the ability to deal with and interact with problems, self-awareness, and love and affection, general thinking and doctrinal dimension, and dealing with moral issues.

Table 3 presents the β values of the effective variables, indicating the priority of affecting communication competence and communication self-efficacy. Moreover, the coefficients of determination of the processed model (R^2^ Adjusted) for communication competence and communication self-efficacy are 0.59 and 0.63, respectively, suggesting that 59% and 63% of the variation in communication competence and self-efficacy scores can be explained by the variables in the model, respectively. The linear equations of nurses’ communication competence and communication self-efficacy score can be explained by the model variables. These equations were obtained according to the multiple regression analysis as follows:

Ya = 2.692 + 0.817X_1_ + 0.774X_2_ + 0.762X_3_ + 0.684X_4_

Yb =  3.547 + 0.876X_1_ + 0.847X_2_ + 0.815X_3_ + 0.808X_4_

Y_a_: nurses’ communication competence score.

Y_b_: nurses’ communication self-efficacy score.

X_1,2,3,4_: Variables affecting communication competence and communication self-efficacy (Table 3).

**Table 3:** Factors affecting communication competence and communication self-efficacy using a multiple linear regression model

Variable			Unstandardized coefficients		Standardized coefficientβ	R^2^ Adjusted	P-value
			B	Std. Error			
**Communication competence**	**---**	(Constant)	2.692	1.402	---	0.59	0.04
	X_1_	The ability to deal with and interact with problems	0.817	0.171	0.725		< 0.001
	X_2_	Self-awareness, love, and affection	0.774	0.166	0.679		< 0.001
	X_3_	General thinking and doctrinal dimension	0.762	0.156	0.671		0.003
	X_4_	Dealing with moral issues	0.684	0.149	0.595		0.005
**Communication self-efficacy**	**---**	(Constant)	3.547	1.926	---	0.63	0.01
	X_1_	The ability to deal with and interact with problems	0.876	0.186	0.796		< 0.001
	X_2_	Self-awareness, love, and affection	0.847	0.181	0.765		< 0.001
	X_3_	General thinking and doctrinal dimension	0.815	0.178	0.734		0.001
	X_4_	Dealing with moral issues	0.808	0.173	0.718		0.004

According to the results, the nurses’ communication competence had a statistically significant relationship with their work experience (*p* = 0.01). In this regard, with an increase in the nurses’ work experience, their mean score of communication competence increased. Furthermore, the mean score of communication competence was significantly different regarding the level of education (*p* = 0.003) and the number of shifts (*p* = 0.01). Moreover, the mean scores of communication competence in nurses with a master’s degree (90.48 ± 7.72 out of 180) and nurses with < 10 shifts per month (91.21 ± 7.61 out of 180) were higher than others. Furthermore, a statistically significant correlation was observed between nurses’ self-efficacy and their work experience (*p* = 0.002). Accordingly, with an increase in the nurses’ work experience, the mean score of their communication self-efficacy increased. Further, significant differences were observed between the mean scores of nurses’ communication self-efficacy regarding gender (*p* = 0.04), level of education (*p* = 0.02), number of shifts (*p* = 0.001), and the number of patients under observation per work shift (*p* = 0.03) as such the mean score of women’s communication self-efficacy (65.36 ± 5.77 out of 120) was higher than that of men. And the mean scores were higher in nurses with a master’s degree (65.82 ± 6.24 out of 120), nurses with < 10 shifts per month (66.53 ± 6.22 out of 120), and those with two patients under observation per work shift (67.35 ± 6.03 out of 120). Finally, there was a significant correlation between the nurses’ SI and their age (*p* = 0.04), and the mean score of this intelligence was different regarding the nurses’ gender (*p* = 0.03). Accordingly, SI in nurses aged 30–40 years (148.22 ± 11.36 out of 210) was higher than in other groups, and female nurses’ mean scores (149.03 ± 11.56 out of 210) were larger than male nurses (Table 4).

**Table 4:** Relationship between communication competence, communication self-efficacy, and SI with the demographic variables among nurses

Variables	Categories	Communication competence		Communication self-efficacy		Spiritual Intelligence (SI)		
		Mean ± SD (From 180)	P-Value	Mean ± SD (From 120)	P-Value	Mean ± SD (From 210)	P-Value	
Age	30>	87.71 ± 6.52	0.09	63.51 ± 5.29	0.10	146.97 ± 11.29		
	30–40	90.56 ± 8.01		68.49 ± 6.12		148.22 ± 11.36	**0.04**	
	40<	89.06 ± 7.11		61.36 ± 5.09		146.19 ± 10.84		
Work experience	10>	86.02 ± 6.75	**0.01**	63.09 ± 4.92	**0.002**	144.01 ± 10.71		
	10–20	90.10 ± 7.44		64.10 ± 5.74		148.11 ± 11.08	0.11	
	20<	91.23 ± 7.12		66.21 ± 5.65		149.27 ± 11.32		
Gender	Male	87.03 ± 6.57	0.12	63.54 ± 5.29	**0.04**	145.23 ± 11.12	**0.03**	
	Female	91.19 ± 7.82		65.36 ± 5.77		149.03 ± 11.56		
Marital status	Single	88.74 ± 6.92	0.08	63.19 ± 5.13	0.13	146.84 ± 10.68	0.14	
	Married	89.48 ± 7.43		65.71 ± 5.78		147.42 ± 11.42		
Level of education	Bachelors’	87.74 ± 7.14	**0.003**	63.08 ± 5.17	**0.02**	146.93 ± 11.19	0.21	
	Master’s	90.48 ± 7.72		65.82 ± 6.24		147.33 ± 11.42		
Type of employment	Formal	91.35 ± 7.32	0.16	66.24 ± 6.38	0.07	148.66 ± 11.62		
	Contractual	89.56 ± 7.32		64.27 ± 5.58		148.38 ± 11.49		
	Contract-Based	88.28 ± 7.32		63.75 ± 5.67		146.65 ± 11.45	0.19	
	Project	88.60 ± 7.32		65.48 ± 5.79		146.59 ± 11.47		
	Corporate	87.98 ± 7.32		62.51 ± 5.43		145.37 ± 11.31		
Number of shifts per month	10>	91.21 ± 7.61	**0.01**	66.53 ± 6.12	**0.001**	149.11 ± 11.56		
	10–20	89.36 ± 7.48		64.42 ± 5.75		147.12 ± 11.38	0.17	
	20<	86.76 ± 6.52		62.40 ± 5.04		145.16 ± 11.72		
Number of patients under observation per work shift	2	92.13 ± 7.26	0.08	67.35 ± 6.03	**0.03**	148.88 ± 11.42		
	3	89.98 ± 7.15		64.18 ± 5.13		148.14 ± 11.32	0.31	
	3<	85.22 ± 6.77		61.82 ± 5.54		144.37 ± 10.55		

## Discussion

This study aimed to detect communication competence and self-efficacy and their relationship with the SI of nurses working in a COVID-19 hospital in Iran. The findings of the present study revealed a moderate level of communication competence among the nurses studied. In contrast to the present study, the communication competence of the nurses in other studies by Najafi-Ghezeljeh et al. [41] in Iran, Hsu et al. [43] in China, and Park et al. [44] in South Korea was above the moderate level. On the other hand, the communication competence of nurses in Lee et al.’s [45] and Son et al.’s [46] studies was insufficient and low. The inconsistencies might have been caused by the tools and the size of the studied population. Although communication is now an integral part of nursing education in most nursing curricula, further studies are necessary to revise educational curricula as such, all nurses with competency levels enter clinical settings.

The findings of this study showed a moderate level of the nurses’ communication self-efficacy. The communication self-efficacy of 234 Iranian nurses [40] and 156 Norwegian nursing students [47] and the general self-efficacy of 214 Korean nurses [44], 175 Cypriot nurses [48], and 143 Polish nurses [49] were above the moderate level, and this is inconsistent with the findings of the present study. The difference in the number and individual characteristics of the research participants may be the main reason for the conflicting results. The present findings indicated the acceptable level of SI in nurses. Studies have revealed different levels for SI in different studies [30, 50–53]. Inconsistencies can be explained by regional cultural values and the tools used to measure SI.

The findings of this study showed a statistically significant and positive correlation between nurses’ communication competence and spiritual intelligence. In line with the present findings, some scholars [34–36] confirm the positive and significant relationship between communication skills and spiritual intelligence. On the other hand, George [39] considers the effectiveness of interpersonal communication as one of the main features of spiritual intelligence. Higher SI increases the likelihood of acceptable and appropriate behaviors such as forgiveness, humility, kindness, patience, compassion, and empathy and creates positive and constructive relationships among individuals. In clinical settings, SI may help nurses to communicate better with their patients. Moreover, nurses with higher SI respond better to stressful situations and can better control situations posing pressure, thus being more successful in communicating effectively with patients and their companions.

The findings of this study also indicated a significant and direct correlation between nurses’ communication self-efficacy and their spiritual intelligence. Similarly, Hatami et al. [30] reported a positive and significant relationship between SI and nurses’ self-efficacy in clinical performance. Beiranvand et al. [32] documented a positive and significant correlation between SI and self-efficacy in nurses. Moreover, a positive and significant relationship between SI and self-efficacy was confirmed by previous studies [29, 31, 33, 38]. These findings are consistent with those of the present study. SI can create a positive attitude in individuals towards themselves, others, and the world; hence, changing the attitude and improving knowledge can improve individuals’ performance and increase their self-efficacy.

Examining the relationship between the main research variables and demographic characteristics showed a positive and significant correlation between communication competence and nurses’ work experience. In this regard, with an increase in work experience, the nurses’ communication competence also increased. In justifying this finding, nurses with more work experience definitely have more experience, and this would help nurses to react appropriately in different situations, listen to patients more carefully, and establish a more appropriate relationship with them. In Park et al.’s [44] study, no significant relationship was observed between communication competence and work experience, which is in contrast with the findings of the present study. The number of participants and different individual characteristics may explain this inconsistency.

Furthermore, the mean scores of communication competence were different regarding the level of education. In this regard, nurses with a master’s degree had higher communication competence. In a study, a positive and significant relationship was found between communication competence and an increased level of education, which is in line with the present study [41]. This finding was expected because an increase in the level of education is likely to improve nurses’ views of patients, and they will become more familiar with the importance of communication in the process of treating and caring for patients; hence, they can communicate more effectively with patients. Unlike the present study, Park et al. [44] found no significant relationship between communication competence and level of education. The inconsistency of the findings is caused by the difference in size and the individual and cultural characteristics of the research subjects.

Moreover, the mean score of communication competence was different regarding the number of shifts per month, so nurses with < 10 shifts per month had a higher level of communication competence. This problem can be caused by the fact that the less fatigue, boredom, and pressure nurses experience, the better they can communicate with patients. In line with the present study, some researchers in Iran reported that with an increase in working hours (number of shifts) and the number of patients, communication competence and effective communication between nurses and patients decreases [41, 51, 54]. According to the findings, nurses’ communication self-efficacy had a positive and significant correlation with their work experience. Moreover, the mean score of communication self-efficacy was significantly higher in female nurses, with a master’s degree, with < 10 shifts per month, and with two patients under observation. Unlike the present study, the studies by Hu et al. [55] in China and Park et al. in South Korea [44] revealed no significant relationship between the nurses’ communication self-efficacy with their work experience and level of education. However, Najafi-Ghezeljeh et al. [41] reported a positive and significant relationship between nurses’ communication self-efficacy with their work experience and level of education and its inverse and significant relationship with working hours (number of shifts), which is in line with the present findings. This implies that the more knowledge, mastery, and experience nurses have in using communication skills, the more self-efficacy they have in communicating with patients.

Finally, a statistically significant and direct correlation was observed between nurses’ SI and age. In this regard, the SI in the age group of 30–40 years was higher than in the nurses with < 30 years of age and > 40 years of age. Moreover, the female nurses’ SI was significantly higher compared to males. Unlike the present study, Imani et al. [56] in Iran found no significant relationship between the nursing students’ mean score of SI and their age and gender. It should be noted that the sample size and tools used in their study were different from the present study. Regarding implications for research and practice, the findings of the present study can provide a concrete evidence for awareness of managers and policy makers in the field of nursing and expanding their knowledge. This study can also be a suitable initial step for planning and developing programs to improve the spiritual intelligence and communication competence and self-efficacy of nurses. In addition, as this study conducted during the covid-19 pandemic, the results of this study can be a solid basis of comparison for future studies as well as the basis of comparison for studies in normal and non-critical situations.

The limitations of this study are the cross-sectional survey, the collection of self-reported information that may have had an effect on the nurses’ reports, the use of a questionnaire, and the non-generalizability of the findings to other societies and cultures. Accordingly, interventional, qualitative, and longitudinal studies with larger sample sizes in research settings with different cultures and in other hospitals are recommended.

## Conclusion

The findings revealed a significant relationship between SI with communication competence, communication self-efficacy, and some demographic characteristics of the nurses. Accordingly, the authorities of teaching-treatment centers and nursing managers are recommended to pay special attention to promoting SI and its dimensions (general thinking and doctrinal dimension, the ability to deal with and interact with problems, self-awareness, and dealing with moral issues) among nurses. They also should provide appropriate interventions such as holding regular training courses in the presence of experienced professors and using various teaching methods to consider SI and its role in interpersonal communication. Additionally, nurses should receive communication skills training to enhance their communication competence and self-efficacy in order to establish effective communication with their supervisors, colleagues, and hospital visitors.

## Data Availability

All the data is presented as a part of tables or figures. Additional data can be requested from the corresponding author.
